# HIV situation in the Greater Mekong Sub-Region bordering Thailand

**DOI:** 10.1186/1742-4690-9-S1-P110

**Published:** 2012-05-25

**Authors:** Tawatchai Apidechkul

**Affiliations:** 1 Professor at Mae Fah Lung University, Chiang Rai Province, Thailand

## Introduction

Greater Mekong Sub-region (GMS) is composing of the border of Thailand-Myanmar, Republic Lao, and China with various of geographic and cultures and more than 200 million people live there. The migration of population is simple scenario at these areas. Last few decades Thailand has been reported of the highest prevalent of HIV/AIDS particularly in the northern of Thailand. The study aimed to investigate the effect of HIV/AIDS at the areas of GMS.

## Materials and methods

This retrospective cohort study design aimed to investigates the HIV situation among the people who immigrated into Thailand in GMS. The systemic data extraction from the medical records from 19 hospitals which located in the border of Thailand during 1990-2009 was analyzed. The instruments had been detected in validity and reliability. Chi-square test was used for identifying the statistical significant at the alpha=0.050.

## Results

Totally 1,303 cases had been detected. Of 84.50% were still alive, 57.27%were male. Myanmar were 56.52%, 26.44% were unknown, and 11.10%were Republic Lao, 5.24%were Chinese, and 1.00% was Cambodian. Of 37.53% were aged 21-30 years old, 33.15% were aged 31-40 years old, and 12.89% were aged 41-50 years old. Of 38.94%were agricultural, 34.56% were employee, 4.15%were student, and 3.46% were young children. Of 51.65% were full bone AIDS, and 28.32 were symptomatic AIDS. Of 92.31% had infected by sexual intercourse, 6.06% were mother to child, and 1.63%wereIDU. Distribution of age by sex was statistical difference (p-value<0.001). Being female had a longer live than male (p-value=0.002). The live status was different according to nationality (p-value<0.001) and risk factor (p-value<0.001).

## Conclusion

The cooperation between countries to control the HIV spreading is immediately need for the GMS region especially the free trade market in 2015.

